# Genome sequence of the pink to light reddish-pigmented *Rubellimicrobium mesophilum* type strain (DSM 19309^T^), a representative of the *Roseobacter* group isolated from soil, and emended description of the species

**DOI:** 10.4056/sigs.5621012

**Published:** 2014-03-20

**Authors:** Thomas Riedel, Stefan Spring, Anne Fiebig, Jörn Petersen, Markus Göker, Hans-Peter Klenk

**Affiliations:** 1 Sorbonne Universités, UPMC Univ Paris 06, USR3579, LBBM, Observatoire Océanologique, Banyuls/Mer, France; 2 CNRS, USR3579, LBBM, Observatoire Océanologique, Banyuls/Mer, France; 3 Leibniz Institute DSMZ – German Collection of Microorganisms and Cell Cultures, Braunschweig, Germany

**Keywords:** Irregular rod-shaped, motile, aerobic, stenohaline, chemoorganotroph, *Roseobacter* group, *Roseobacter* group, *Rhodobacteraceae*, *Alphaproteobacteria*

## Abstract

*Rubellimicrobium mesophilum* Dastager *et al.* 2008 is a mesophilic and light reddish-pigmented representative of the *Roseobacter* group within the alphaproteobacterial family *Rhodobacteraceae*. Representatives of the *Roseobacter* group play an important role in the marine biogeochemical cycles and were found in a broad variety of marine environments associated with algal blooms, different kinds of sediments, and surfaces of invertebrates and vertebrates. Roseobacters were shown to be widely distributed, especially within the total bacterial community found in coastal waters, as well as in mixed water layers of the open ocean. Here we describe the features of *R. mesophilum* strain MSL-20^T^ together with its genome sequence and annotation generated from a culture of DSM 19309^T^. The 4,927,676 bp genome sequence consists of one chromosome and probably one extrachromosomal element. It contains 5,082 protein-coding genes and 56 RNA genes. As previously reported, the G+C content is significantly different from the actual genome sequence-based G+C content and as the type strain tests positively for oxidase, the species description is emended accordingly. The genome was sequenced as part of the activities of the Transregional Collaborative Research Centre 51 (TRR51) funded by the German Research Foundation (DFG).

## Introduction

Strain MSL-20^T^ (= DSM 19309^T^ = KCTC 22012^T^) is the type strain of the species *Rubellimicrobium mesophilum* [1], one of four species with validly published names in the genus *Rubellimicrobium* [2,3]; the other three species in the genus are *R. thermophilum* [3], *R. aerolatum* [4] and *R. roseum* [5]. *Rubellimicrobium* belongs to the abundant marine *Roseobacter* group [6]. The species epithet *mesophilum* refers to the Greek adjective *mesos*, middle, as well as from the Neo-Latin adjective ‘*philus –a –um*’, friend/loving [1], the middle (temperature-) loving. Strain MSL-20^T^ was isolated from soil located at Bigeum Island, Republic of Korea [1], whereas the other type strains within the genus *Rubellimicrobium* were isolated from a paper mill (*R. thermophilum* [3]), air (*R. aerolatum* [4]) and forest soil (*R. roseum* [5]), which indicates rather diverse habitats for *Rubellimicrobium*. Current PubMed records do not indicate any follow-up research with strain MSL-20^T^ since the initial description of *R. mesophilum* [1]. Here we present a summary classification and a set of features for *R. mesophilum* MSL-20^T^, together with the description of the complete genomic sequencing and annotation.

## Classification and features

### 16S rRNA gene analysis

**Figure 1:**
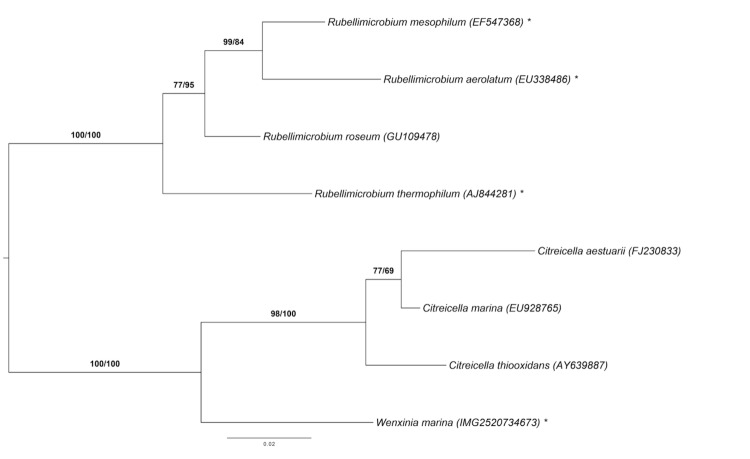
Phylogenetic tree highlighting the position of *R. mesophilum* relative to the type strains of the other species within the genus *Rubellimicrobium* and the neighboring genera *Citreicella* and *Wenxinia*. The tree was inferred from 1,381 aligned characters of the 16S rRNA gene sequences under the maximum likelihood (ML) criterion as previously described [7]. The branches are scaled in terms of the expected number of substitutions per site. Numbers adjacent to the branches are support values from 1,000 ML bootstrap replicates (left) and from 1,000 maximum-parsimony bootstrap replicates (right) if larger than 60% [7]. Lineages with type strain genome sequencing projects registered in GOLD [8] are labeled with one asterisk [9].

Figure 1 shows the phylogenetic neighborhood of *R. mesophilum* in a 16S rRNA gene sequence-based tree. The sequence of the single 16S rRNA gene in the DSM 19309^T^ genome does not differ from the previously published 16S rRNA gene sequence (EF547368), which contains four ambiguous base calls.

The genomic 16S rRNA gene sequence of *R. mesophilum* DSM 19309^T^ was compared with the Greengenes database for determining the weighted relative frequencies of taxa and (truncated) keywords as previously described [7]. The most frequently occurring genera were *Paracoccus* (45.3%), *Loktanella* (30.3%), *Rubellimicrobium* (14.0%), *Methylarcula* (8.4%) and *'Pararubellimicrobium'* (2.0%) (58 hits in total). Regarding the five hits to sequences from other members of the genus, the average identity within HSPs was 94.9%, whereas the average coverage by HSPs was 99.3%. Among all other species, the one yielding the highest score was *'Pararubellimicrobium aerilata'* (EU338486), which corresponded to an identity of 96.3% and a HSP coverage of 98.0%. (Note that the Greengenes database uses the INSDC (=EMBL/NCBI/DDBJ) annotation, which is not an authoritative source for nomenclature or classification). The highest-scoring environmental sequence was JF417792 (Greengenes short name 'microbial structures coalbeds located Eerduosi Basin China coalbed clone QQSB73'), which showed an identity of 98.7% and a HSP coverage of 99.6%. The most frequently occurring keywords within the labels of all environmental samples which yielded hits were 'skin' (10.6%), 'fossa' (5.9%), 'poplit' (4.2%), 'forearm, volar' (3.3%) and 'sea' (2.8%) (192 hits in total). Environmental samples which yielded hits of a higher score than the highest scoring species were not found, indicating that *R. mesophilum* has rarely been detected in the environment.

### Morphology and physiology

**Figure 2:**
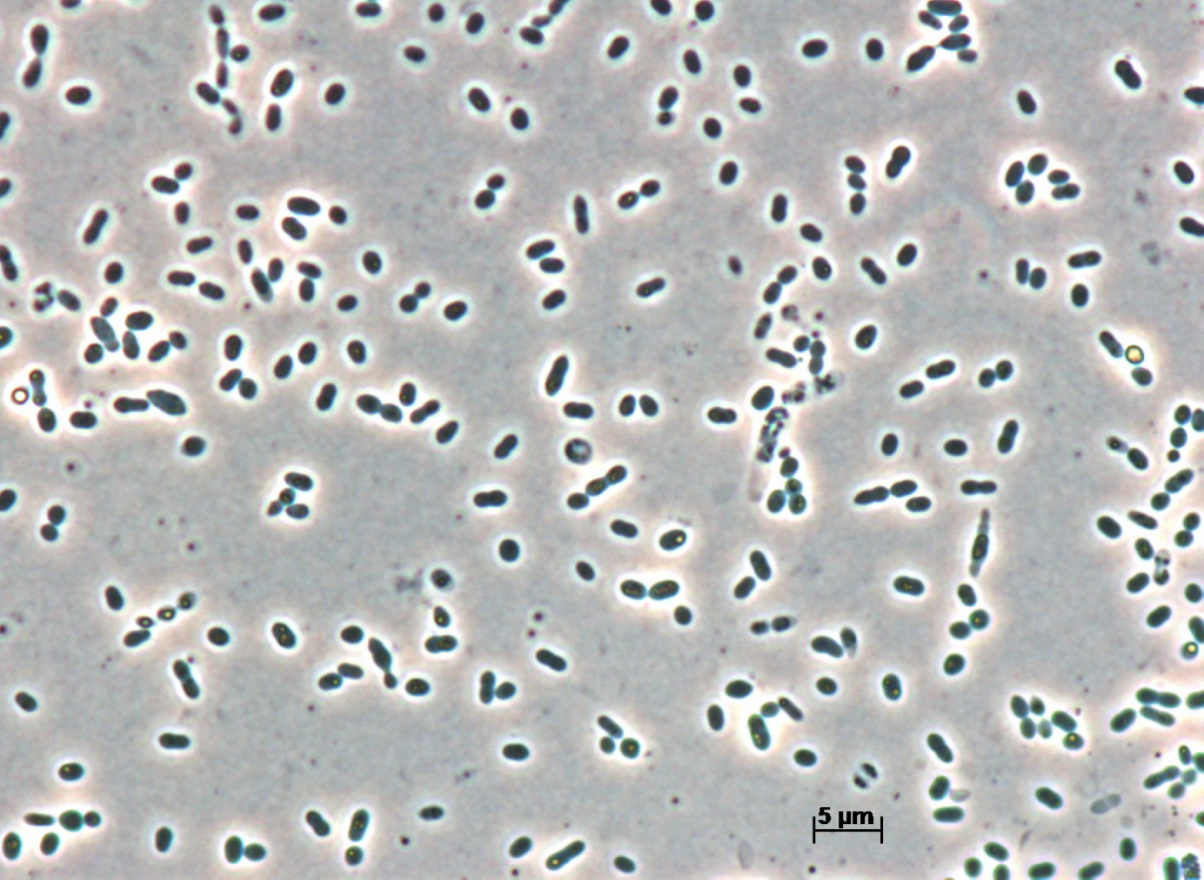
Micrograph of *R. mesophilum* DSM 19309^T^.

Cells of strain MSL-20^T^ stain Gram-negative, are described to be motile (without a flagellum) [1], and ovoid or rod-shaped, 1.6-3.4 µm in length and 0.4-0.7 µm in width (Figure 2 and Table 1). On Reasoner’s 2A (R2A) agar they form pink to light red-pigmented colonies. According to [1], cells are negative for oxidase (but see below) and nitrate reduction activities, but show only weak catalase activity. They hydrolyze starch and Tween 80, assimilate cellulose, histidine, leucine and fructose, but do not utilize citrate and propionate. Cells test positive for leucine arylamidase, naphthol-AS-BI-phosphohydrolase and *α*-glucosidase. Growth is observed in a temperature range of 20-37°C with an optimum at 28°C. The pH range for growth is between pH 7-11 with an optimum at pH 7.0 ± 0.2. No growth occurs in the presence of NaCl in concentrations of 0.5% and above. Cells of strain MSL-20^T^ do not utilize the carbohydrates cellobiose, D-mannose, salicin, D-xylose, α-melibiose, D-sorbitol, L-malate and D-ribose, which are utilized by its close relative *R. thermophilum* DSM 16684^T^ (all data from [1]).

**Table 1:** Classification and general features of *R. mesophilum* MSL-20^T^ according the MIGS recommendations [10] published by the Genome Standards Consortium [11].

**MIGS ID**	**Property**	**Term**	**Evidence code**
	Current classification	Domain *Bacteria*	TAS [12]
		Phylum *Proteobacteria*	TAS [13]
		Class *Alphaproteobacteria*	TAS [14,15]
		Order *Rhodobacterales*	TAS [15,16]
		Family *Rhodobacteraceae*	TAS [15,17]
		Genus *Rubellimicrobium*	TAS [3]
		Species *Rubellimicrobium mesophilum*	TAS [1]
		Strain MSL-20^T^	TAS [1]
	Gram stain	negative	TAS [1]
	Cell shape	irregular rod-shaped	TAS [1]
	Motility	motile	TAS [1]
	Sporulation	non-sporulating	NAS
	Temperature range	20-37°C	TAS [1]
	Optimum temperature	28°C	TAS [1]
	Salinity	stenohaline	TAS [1]
MIGS-22	Oxygen requirement	aerobic	TAS [1]
	Carbon source	carbohydrates, amino acids	TAS [1]
	Energy metabolism	chemoorganotroph	NAS
MIGS-6	Habitat	soil	TAS [1]
MIGS-15	Biotic relationship	free living	TAS [1]
MIGS-14	Pathogenicity	none	NAS
	Biosafety level	1	TAS [18]
MIGS-23.1	Isolation	soil	TAS [1]
MIGS-4	Geographic location	Bigeum island (Republic of Korea)	TAS [1]
MIGS-5	Sample collection time	April 2006	NAS
MIGS-4.1	Latitude	34.739	NAS
MIGS-4.2	Longitude	125.920	NAS
MIGS-4.3	Depth	not reported	
MIGS-4.4	Altitude	not reported	

Evidence codes - TAS: Traceable Author Statement (i.e., a direct report exists in the literature); NAS: Non-traceable Author Statement (i.e., not directly observed for the living, isolated sample, but based on a generally accepted property for the species, or anecdotal evidence). Evidence codes are from of the Gene Ontology project [19].

### Chemotaxonomy

The principal cellular fatty acids of strain MSL-20^T^ are C_16:0_ (36.9%), C_18:1 ω7c_ (36.5%), 11-methyl C_18:1 ω7c_ (12.4%), C_18:0_ (3.6%), C_10:0_ (1.3%), C_12:0_ (1.3%) and C_17:0_ (1.2%) and differ significantly from those detected in *R. thermophilum*. The major respiratory lipoquinone is ubiquinone Q-10, which is a common feature of alphaproteobacterial representatives (all data from [1]).

## Genome sequencing and annotation

### Genome project history

The genome of strain *R. mesophilum* DSM 19309^T^ was first selected for genome sequencing in phase I of the one thousand microbial genomes (KMG-I) project [20], an extension of the Genomic Encyclopaedia of *Bacteria* and *Archaea* (GEBA) [21], but ultimately sequenced within the DFG funded project “Ecology, Physiology and Molecular Biology of the *Roseobacter* clade: Towards a Systems Biology Understanding of a globally Important Clade of Marine Bacteria”. The strain was chosen for genome sequencing according to a phylogeny-driven target selection procedure for large scale genome-sequencing (and other) projects as routinely used for the KMG-I project [20,22].

The project information can be found in the Genome OnLine Database [8]. The Whole Genome Shotgun (WGS) sequence is deposited in GenBank and the Integrated Microbial Genomes database (IMG) [23]. A summary of the project information is shown in Table 2.

**Table 2:** Genome sequencing project information

MIGS ID	Property	Term
MIGS-31	Finishing quality	Non-contiguous finished
MIGS-28	Libraries used	Two genomic libraries: one Illumina PE library (420 bp insert size), one 454 PE library (3 kb insert size)
MIGS-29	Sequencing platforms	Illumina GA II×, Illumina MiSeq, 454 GS-FLX+Titanium
MIGS-31.2	Sequencing coverage	129×
MIGS-30	Assemblers	Velvet version 1.1.36, Newbler version 2.3, Consed 20.0
MIGS-32	Gene calling method	Prodigal 1.4
	INSDC ID	AOSK00000000
	GenBank Date of Release	*pending publication*
	GOLD ID	Gi0042374
	NCBI project ID	188767
	Database: IMG	2523533591
MIGS-13	Source material identifier	DSM 19309^T^
	Project relevance	Tree of Life, biodiversity

### Growth conditions and DNA isolation

A culture of DSM 19309^T^ was grown aerobically in DSMZ medium 830 (R2A medium) [24] at 28°C. Genomic DNA was isolated using Jetflex Genomic DNA Purification Kit (GENOMED 600100) following the standard protocol provided by the manufacturer, but modified by an incubation time of 60 min, an overnight incubation on ice on a shaker, the use of additional 50 µl proteinase K, and the addition of 100 µl protein precipitation buffer. DNA is available from DSMZ through the DNA Bank Network [25].

### Genome sequencing and assembly

The genome was sequenced using a combination of two libraries (Table 2). The paired-end library contained inserts of an average of 420 bp in length. Illumina sequencing was performed on a GA IIx platform with 150 cycles. The first run on the Illumina GA IIx platform delivered 3.6 million reads. In order to increase the sequencing depth, a second Illumina run was performed, providing another 7.0 million reads. Error correction and clipping were performed by fastq-mcf [26] and quake [27]. The data was assembled using Velvet [28]. The first draft assembly from 5,400,234 filtered reads (median read length of 132 nt) resulted in more than 143 unordered contigs. To gain information about the contig arrangement an additional 454 run was performed. The paired-end jumping library of 3 kb insert size was sequenced on 1/8 of a lane. Pyrosequencing resulted in 102,695 reads with an average read length of 199 bp, assembled with Newbler (Roche Diagnostics). The resulting assembly consisted of 261 scaffolds. Both draft assemblies (Illumina and 454 sequences) were fractionated into artificial Sanger reads of 1,000 nt in length plus 75 bp overlap on each site. These artificial reads served as an input for the phred/phrap/consed package [29]. By manual editing, 138 contigs could be assembled on 127 scaffolds. The combined sequences provided a 129× coverage of the genome.

### Genome annotation

Genes were identified using Prodigal [30] as part of the JGI genome annotation pipeline. The predicted CDSs were translated and used to search the National Center for Biotechnology Information (NCBI) non-redundant database, UniProt, TIGR-Fam, Pfam, PRIAM, KEGG, COG, and InterPro databases. Identification of RNA genes was carried out by using HMMER 3.0rc1 [31] (rRNAs) and tRNAscan-SE 1.23 [32] (tRNAs). Other non-coding genes were predicted using INFERNAL 1.0.2 [33]. Additional gene prediction analysis and functional annotation was performed within the Integrated Microbial Genomes - Expert Review (IMG-ER) platform [34]. CRISPR elements were detected using CRT [35] and PILER-CR [36].

## Genome properties

**Figure 3:**
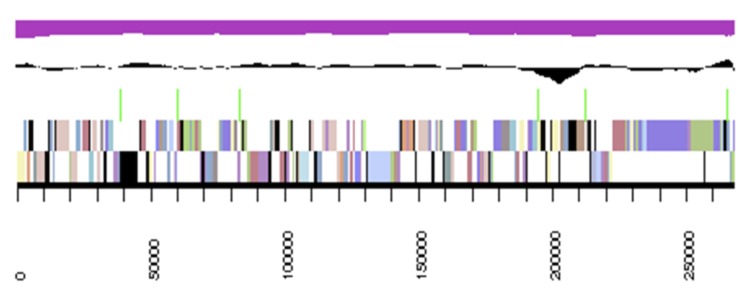
Graphical map of the largest, 267,932 bp long scaffold. From bottom to the top: Genes on forward strand (colored by COG categories), Genes on reverse strand (colored by COG categories), RNA genes (tRNAs green), GC content (black), GC skew (purple/olive).

The genome statistics are provided in Table 3 and Figure 3. The genome of strain DSM 19309^T^ has a total length of 4,927,676 bp and a G+C content of 69.7%. Of the 5,138 genes predicted, 5,082 were identified as protein-coding genes, and 56 as RNAs. The majority of the protein-coding genes (56.7%) were assigned a putative function while the remaining ones were annotated as hypothetical proteins. The distribution of genes into COGs functional categories is presented in Table 4.

**Table 3:** Genome statistics

**Attribute**	Value	% of Total
Genome size (bp)	4,927,676	100.00
DNA coding region (bp)	4,254,404	86.34
DNA G+C content (bp)	3,431,981	69.65
Number of scaffolds MIGS-9	127	
Extrachromosomal elements MIGS-10	1	
Total genes	5,138	100.00
RNA genes	56	1.09
rRNA operons	1	
tRNA genes	45	0.88
Protein-coding genes	2,915	56.73
Genes with function prediction (proteins)	2,167	42.18
Genes in paralog clusters	4,172	81.20
Genes assigned to COGs	3,818	74.31
Genes assigned Pfam domains	3,977	77.40
Genes with signal peptides	384	7.47
Genes with transmembrane helices	966	18.80
CRISPR repeats	0	

**Table 4:** Number of genes associated with the general COG functional categories

**Code**	**Value**	**%age**	**Description**
J	186	4.4	Translation, ribosomal structure and biogenesis
A	3	0.1	RNA processing and modification
K	279	6.6	Transcription
L	250	5.9	Replication, recombination and repair
B	4	0.1	Chromatin structure and dynamics
D	35	0.8	Cell cycle control, cell division, chromosome partitioning
Y	0	0.0	Nuclear structure
V	34	0.8	Defense mechanisms
T	176	4.2	Signal transduction mechanisms
M	223	5.3	Cell wall/membrane/envelope biogenesis
N	27*	0.6	Cell motility
Z	0	0.0	Cytoskeleton
W	0	0.0	Extracellular structures
U	51	1.2	Intracellular trafficking and secretion, and vesicular transport
O	148	3.5	Posttranslational modification, protein turnover, chaperones
C	251	6.0	Energy production and conversion
G	453	10.7	Carbohydrate transport and metabolism
E	478	11.3	Amino acid transport and metabolism
F	94	2.2	Nucleotide transport and metabolism
H	152	3.6	Coenzyme transport and metabolism
I	143	3.4	Lipid transport and metabolism
P	182	4.3	Inorganic ion transport and metabolism
Q	124	2.9	Secondary metabolites biosynthesis, transport and catabolism
R	508	12.0	General function prediction only
S	421	10.0	Function unknown
-	1,320	25.7	Not in COGs

*Only one gene each for flagellar motor and flagellar hook capping, no structural genes for flagella.

## Insights into the genome

### Plasmids

The identification of plasmids is difficult because typical replication modules comprising the characteristic replicase and the adjacent *parAB* partitioning operon are missing [36]. However, comprehensive BLASTP searches with plasmid replicases from *Rhodobacterales* revealed the presence of one RepB gene (rumeso_01479), whereas RepA-, RepABC-type and DnaA-like replicases are absent from the genome. The localization of the chromosomal replication initiator DnaA documents that scaffold 15 is part of the chromosome (Table 5).

**Table 5:** General genomic location and features of the chromosomal and one extrachromosomal replicon from *R. mesophilum* strain DSM 19309^T^.

**Replicon**	**Scaffold**	**Replicase**	**Length** (bp)	**GC** (%)	**Topology**	**No. Genes^#^**
Chromosome^1^	15	DnaA	102,082	71	linear*	105
Plasmid	9	RepB	119,205	68	linear*	141

*circularity not experimentally validated

^#^deduced from automatic annotation

^1^partial sequence including the replicase *dnaA* (rumeso_02152).

The 119 kb RepB type plasmid contains a post-segregational killing system (PSK) consisting of a typical operon with two small genes encoding a stable toxin and an unstable antitoxin (rumesco_01477/78 [37];).

### Phages

Phages are widely distributed and abundant in marine and freshwater environments [38-40] and are known to be horizontal gene transfer agents that drive bacterial diversity [40,41]. Temperate phage genomes can be integrated in the host genome as prophages and perform a symbiotic relationship with their hosts [42].

Several phage-associated gene sequences were detected in the genome sequence of *strain* DSM 19309^T^, particularly in “genomic islands” (e.g., rumeso_00405, rumeso_00407 rumeso_01586 to rumeso_01600).

### Quorum Sensing

Several Gram-negative bacteria produce and release chemical signal molecules called autoinducers. In correlation to the population density they detect those signal molecules and respond with an alteration of gene expression and therefore with diverse behaviors (e.g., luminescence, virulence, antibiotic resistance, changes in morphology and cell division) [43-46].

Genome analysis of strain DSM 19309^T^ revealed the presence of gene-encoding sequences associated with the mechanism of quorum sensing e.g. N-homoserine-lactone synthetase, rumeso_02218 (*LuxI* homologue); probably involved in response and transcriptional regulators, rumeso_02217 (*luxR* homologue).

### Metabolic plasticity

Unlike many representatives of the *Roseobacter* group [6], *R. mesophilum* DSM 19309^T^ encodes no genes involved in the harvesting of light and photoheterotrophic growth, which reflect its occurrence in niches within soil that are characterized by the absence of light. Nevertheless, the annotated genome sequence reveals a high metabolic versatility that was not expected by the phenotypic characterization presented in the species description [1].

The genome encodes a large number of diverse ABC transporters facilitating the uptake of various substrates like carbohydrates (e.g., rumeso_04497 to 04500), polyamines (e.g., rumeso_04716 to 04719), peptides (e.g., rumeso_00087 to 00090), amino acids (e.g., rumeso_00231 to 00234) and sulfonates (e.g., rumeso_05058 to 05059). Sulfonates could represent unexpected but common substrates for this species. The organic sulfonates taurine and cysteic acid are widely distributed in animal tissue and can enter soil by feces. In some soil bacteria, these compounds are used as sole source of carbon, nitrogen and sulfur [47]. Indeed, a complete degradation pathway for taurine was detected in the genome of strain DSM 19309^T^. Taurine is first converted by a taurine-pyruvate aminotransferase (rumeso_05057) to sulfoacetaldehyde, which in turn is cleaved by the enzyme sulfoacetaldehyde acetyltransferase (rumeso_03970) into sulfite and acetyl-phosphate. Acetyl-phosphate can be either converted to acetyl-CoA by a phosphotransacetylase (rumeso_03968) and funneled into the intermediary metabolism or is used for the generation of ATP by the enzyme acetate kinase (rumeso_03967). The potentially toxic compound sulfite can be oxidized to sulfate by various sulfite oxidases (e.g., rumeso_03951).

In addition, the utilization of electron acceptors seems to be variable and not restricted to oxygen. Genes encoding at least two predicted cytochrome *c* oxidases, one of the *cbb*_3_-type (rumeso_00470 to 00472) and the other of the *aa*_3_-type (rumeso_02204 to 02206), which terminate the electron transport chain with oxygen, were detected. However, according to the species description strain MSL-20^T^ should be oxidase negative [1], we have found that the oxidase test for this strain is positive, which is in line with the results of the genome analysis.

Under periodic anoxic conditions that frequently occur in wet soils, nitrate could be used as alternative electron acceptor. According to the genome sequence, the denitrification pathway of this strain is probably incomplete and terminates with the greenhouse gas nitrous oxide (N_2_O), as has been previously demonstrated for *Ottowia thiooxydans* [48]. Only genes encoding a respiratory nitrate reductase (rumeso_02471 to 02474), nitrite reductase (rumeso_02669) and nitric oxide reductase (rumeso_00142 to 00145) were detected, whereas no genes for the terminal nitrous oxide reductase were found.

### Comparison of *Rubellimicrobium* genomes

Recently the genome sequence of the type strain for second representative of the genus *Rubellimicrobium*, *R. thermophilum* DSM 16684^T^ became available [9]. Lifestyle, habitat and preferred temperature range of *R. thermophilum* differ significantly from the ones of *R. mesophilum* [3]. The genome sequences of both strains were compared using the digital DNA-DNA hybridization (dDDH) tool GGDC server version 2.0, an online tool provided through the DSMZ web pages [49]. The resulting dDDH value of 19.3 ± 2.3% according to distance formula 2 (as described in [50]), confirmed that both strains belong to independent species.

**Figure 4:**
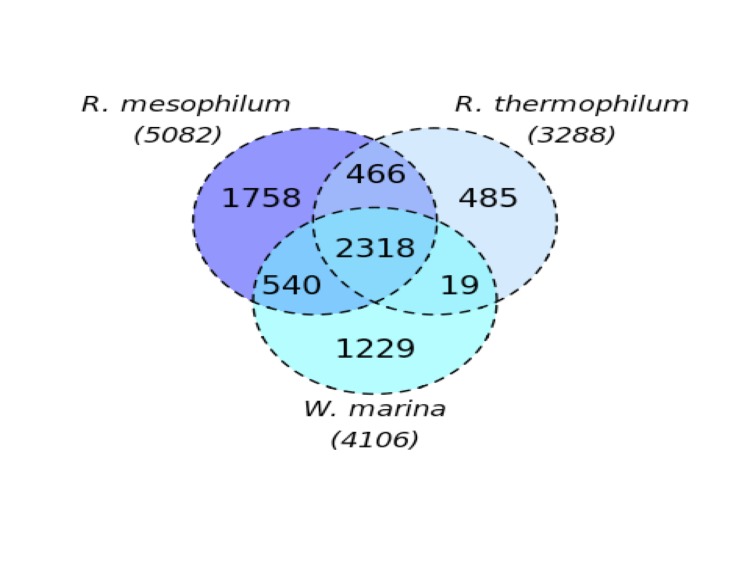
Venn diagram depicting the intersections of proteins sets (total numbers in parentheses) of the two *Rubellimicrobium* species and *W. marina*.

Figure 4 depicts the fraction of shared genes between the two genome-sequenced *Rubellimicrobium* type strains and the type strain of *Wenxinia marina* [51], another closely related member of the *Roseobacter* group (see Figure 1). The number of pairwise genes was inferred from the phylogenetic profiler tool of the IMG platform. Homologous genes were detected with an E-value cutoff of 10^-5^ and a minimum identity of 30%. Proportions of 56% and 45% of the gene count in *W. marina* and *R. mesophilum*, respectively, are shared between all three genomes. In the case of *R. thermophilum,* a fraction of homologous genes of 70% is present in the other two genomes. Very few genes are shared only between *R. thermophilum* and *W. marina*.

Although both genomes differ significantly in size (3.2 Mbp for *R. mesophilum* and 4.9 Mbp for *R. thermophilum*), the proportions of genes per COG category is very similar (Table 3 and [9]). The IMG Abundance Profile [34] demonstrated some differences, however. Enzymes for transport and utilization of amino acids and polyamines (COG1173, COG0747, COG3842) were present in higher abundance in *R. thermophilum*, which is in agreement with the results from wet-lab substrate tests [1,3]. Huge differences in the abundance of proteins can be found within the class of transposases (COG2801, COG 3436, COG2936, COG0665, COG0404). While *R. thermophilum* codes for two transposases, more than 30 tranposase genes were identified in *R. mesophilum*. Combined with the presence of the site-specific recombinase XerD (involved in the recombination of plasmids [20]) this indicates a high level of genetic recombination within *R. mesophilum*. Furthermore, 23 genes coding for RTX toxins and Ca^+^-binding proteins (COG 2931) were found. These proteins are structurally diverse, playing an important role in the colonization of various habitats and surfaces [50]. Additionally, 14 proteins of the xenobiotic-degrading glutathion-S-transferases were present in *R. mesophilum*. The occurrence of these proteins may enable the bacteria to grow in polluted areas.

### Taxonomic note

The G +C content of the genomic DNA of strain MSL-20^T^ is given in the species description as 72.3 mol% [1], which represents a discrepancy of more than 2% from the value of 69.7 mol% deduced from the genome sequence. In addition to the deviant oxidase test this calls for an emendation of the species description according to the proposal of Meier-Kolthoff et al. [47].

## Emended description of the species *Rubellimicrobium mesophilum* Dastager *et al.* 2008

The description of the species *Rubellimicrobium mesophilum* is the one given by Dastager *et al.* 2008 [1], with the following modifications.

Oxidase test is positive. The G+C content, rounded to zero decimal places, is 70%.
